# Association of *SCN10A* Polymorphisms with the Recurrence of Atrial Fibrillation after Catheter Ablation in a Chinese Han Population

**DOI:** 10.1038/srep44003

**Published:** 2017-03-10

**Authors:** Haiqing Wu, Juan Xu, Songwen Chen, Genqing Zhou, Baozhen Qi, Yong Wei, En Hu, Dongdong Tang, Gang Chen, Hongli Li, Liqun Zhao, Yongyong Shi, Shaowen Liu

**Affiliations:** 1 Department of Cardiology, Shanghai First People’s Hospital, Shanghai Jiao Tong University School of Medicine, Shanghai, 200080, China; 2 Department of Cardiology, Zhongshan Hospital, Fudan University, Shanghai, 200032, China; 3 Department of Cardiology, Shanghai Songjiang Center Hospital, Shanghai, 201600, China; 4 Bio-X Institutes, Key Laboratory for the Genetics of Developmental and Neuropsychiatric Disorders (Ministry of Education), Shanghai Jiao Tong University, Shanghai, 200030, China

## Abstract

The nonsynonymous *SCN10A* single nucleotide polymorphism (SNP) rs6795970 has been reported to associate with PR interval and atrial fibrillation (AF) and in strong linkage disequilibrium (LD) with the AF-associated SNP rs6800541. In this study, we investigated whether rs6795970 polymorphisms are associated with AF recurrence after catheter ablation. A total of 502 consecutive patients with AF who underwent catheter ablation were included. AF recurrence was defined as a documented episode of any atrial arrhythmias lasting ≥30 s after a blanking period of 3 months. AF recurrence was observed between 3 and 12 months after catheter ablation in 24.5% of the patients. There was a significant difference in the allele distribution (p = 7.86 × 10^−5^) and genotype distribution (p = 1.42 × 10^−5^) of rs6795970 between the AF recurrence and no recurrence groups. In a multivariate analysis, we identified the following independent predictors of AF recurrence: the rs6795970 genotypes in an additive model (OR 0.36, 95%CI 0.22~0.60, p = 7.04 × 10^−5^), a history of AF ≥36 months (OR 3.57, 95%CI 2.26~5.63, p = 4.33 × 10^−8^) and left atrial diameter (LAD) ≥40 mm (OR 1.85, 95%CI 1.08~3.19, p = 0.026). These data suggest that genetic variation in *SCN10A* may play an important role in predicting AF recurrence after catheter ablation in the Chinese Han population.

Atrial fibrillation (AF) is the most common form of arrhythmia and has been independently associated with an increased risk of stroke, heart failure and death^1^. The current AF guidelines recommend catheter ablation in patients with symptomatic, antiarrhythmic drug refractory AF. Moreover, catheter ablation is suggested as a first-line treatment if there is no or minimal heart disease^2^,^3^. It has been reported that single-procedure ablation achieves freedom from AF in 57 to 89% of patients^4^,^5^ and that results are dependent on patient characteristics, ablation strategies and follow-up times. Although a recent meta-analysis demonstrated that increased left atrial diameter (LAD), non-paroxysmal AF and valvular AF were independent predictors of recurrent AF^6^, it remains challenging to predict outcomes in AF patients who undergo catheter ablation.

Voltage-gated sodium (Na_v_) channels play an important role during the rising phase of action potential (AP) and are critical for impulse generation and conduction in a majority of excitable cells. Na_v_1.5 (encoded by *SCN5A*) is an essential sodium channel that is responsible for regulating cardiac conduction. However, other sodium channel isoforms are also present in the heart. These include Na_v_1.1, Na_v_1.3, Na_v_1.6, Na_v_1.8 and Na_x_^7^,^8^,^9^,^10^. Na_v_1.8 (encoded by *SCN10A*) is a tetrodotoxin (TTX)-resistant sodium channel that is expressed at high levels in the small-diameter sensory neurons of the dorsal root ganglia and cranial sensory ganglia^11^,^12^. Na_v_1.8 was also found in a recent study to be expressd in mouse intrinsic cardiac ganglia^8^. Some GWAS have shown that *SCN10A* is associated with cardiac conduction, as it increases PR interval and QRS duration on the electrocardiogram^13^,^14^,^15^,^16^. Moreover, *SCN10A* has been reported to associated with AF^16^,^17^. Chambers *et al*.^13^ demonstrated that the nonsynonymous *SCN10A* SNP rs6795970 was associated with PR interval. The rs6795970 (G > A) is a missense mutation that causes an A1073V amino acid change within the IDII/III intracellular loop of Na_v_1.8. Ritchie *et al*.^16^ found that rs6795970 was associated with development of AF. Pfeufer *et al*.^17^ showed that rs6800541 in *SCN10A* was significantly associated with AF risk. Moreover, rs6800541 is in strong linkage disequilibrium (LD) with rs6795970 (r^2^ = 0.933).

Nevertheless, the data regarding the relationship between genetic variations and outcomes in AF patients who undergo ablation are currently limited^18^,^19^,^20^,^21^,^22^,^23^. The primary aim of this study was to determine whether rs6795970 polymorphisms are associated with AF recurrence after catheter ablation. In addition, we performed a secondary analysis using a tag-SNP approach to investigate whether there are additional associations in *SCN10A*.

## Methods

### Study population

The current study was reviewed and approved by the Institutional Review Board of Shanghai First People’s Hospital affiliated to Shanghai Jiao Tong University School of Medicine. This study was conducted in accordance with the principles of the Declaration of Helsinki. Written informed consent was obtained from all subjects. Initially, a total of 572 consecutive patients with drug-refractory AF who underwent their first radiofrequency catheter ablation procedure at the Department of Cardiology in Shanghai First People’s Hospital were recruited to the study between April 2011 and February 2014. Paroxysmal AF was defined according to a previously published expert consensus statement^24^. Patients were excluded from the study if (1) they had received a previous endovascular or surgical AF ablation or a maze surgery, (2) they had untreated hyperthyroidism, (3) they had severe pulmonary disease, or (4) they had severe hepatic and renal dysfunction. Information including patient characteristics, clinical data before ablation, ablation records and follow-up data were obtained from our medical database. Finally, a total of 502 patients completed follow-up and were analysed in this study. A transthoracic and transoesophageal echocardiography was performed in all patients before the procedure to exclude a left atrial thrombus. Moreover, standardized measurements were obtained for LAD and left ventricular ejection fraction (LVEF). All class I or III antiarrhythmic medications except amiodarone were discontinued at least 5 half-lives before the ablation was performed. LAD was defined as the left atrial anterior-posterior diameter at the end of cardiac systole, which was measured as the cross-section of the long-axis of the left ventricle adjacent to the sternum. The normal range for LAD was 19–40 mm. History of AF was defined as the time course from the first discovery of AF to the first catheter ablation procedure performed to treat AF in our hospital.

### Preoperative preparation and ablation procedure

The preoperative preparation procedures are described in detail in our previous publications^25^,^26^. The radiofrequency catheter ablation procedure was performed while the AF patients were under sedation (via a bolus of midazolam) and analgesia (via a continuous infusion of fentanyl)^25^. The protocol for AF ablation was previously described in detail^25^,^26^. A multipolar electrode 6 F catheter was placed in the coronary sinus. We performed a transseptal puncture and placed two long sheaths in the left atrium (LA). LA reconstruction was performed using a 3-dimensional mapping system (CARTO; Biosense Webster Inc.). Electroanatomical mapping and ablation were performed using a 3.5-mm-tip catheter (ThermoCool Navi-Star, Biosense Webster, Diamond Bar, CA, USA). Images were integrated using the reconstructed computed tomography scan. The first step of the ablation strategy was to perform circumferential pulmonary veins (PV) ablation. We placed a circular mapping catheter (Lasso, Biosense Webster) within the superior or inferior PV or within the branches of a common PV to identify the breakthrough region for the LA to PV conduction and to guide the gap ablation during PV isolation. If AF persisted, linear ablations (e.g., the left atrial roof, the basal posterior wall and the left atrial isthmus) and complex fractionated atrial electrograms (CFAEs) ablations were performed when necessary. If AF converted into intermediate atrial flutter (AT), we conducted entrainment mapping and activation mapping to identify the mechanism contributing to AT. Ablation was then performed at the critical isthmus or arrhythmogenic focus to terminate AT. Electrical or drug cardioversion was performed to restore sinus rhythm (SR) when AF/AT termination could not be achieved using the abovementioned ablation steps. After cardioversion, we verified the bidirectional conduction block of all ablation lines. If necessary, reinforcement ablation was performed to ensure the bidirectional conduction block. In paroxysmal AF patients, the endpoints included PV isolation and non-inducible atrial arrhythmias lasting for 5 minutes. In non-paroxysmal AF patients, the endpoints were the termination of AF and conversion to a sinus rhythm, including PV isolation. All ablations were performed by the same electrophysiological team, which was led by Professor Shaowen Liu.

Irrigated radiofrequency energy was delivered at a maximum temperature of 4 °C, a maximum radiofrequency power of 38 W and an infusion rate of 17–25 ml/min. In all patients, the upper radiofrequency power limit that was delivered to the superior vena cava and the coronary sinus was set at 25 W, to reduce the risk of cardiac tamponade or phrenic nerve impairment. The power delivered to the posterior wall was limited to 35 W, to reduce the risk of oesophageal injury.

### Follow-up

After the ablation, all patients were followed up in the outpatient clinic for 12 months. Amiodarone or propafenone were administered after ablation if not contraindicated to prevent the early recurrence of AF. Patients without atrial arrhythmia recurrence discontinued amiodarone and propafenone at 3 months after the procedure. Anticoagulation treatment was prescribed for at least 3 months and thereafter according to the CHA_2_DS_2_-VASc score. Proton pump inhibitors were prescribed for 4 weeks. AF recurrence was detected using a 24-hour Holter monitor at 3, 6, and 12 months after the procedure. When a patient’s symptoms became suggestive of AF, supplemental electrocardiograms and a 24-hour Holter monitor were performed. AF recurrence was defined as a documented episode of any atrial arrhythmias (AF, AT and atrial tachycardia) lasting ≥30 s during the follow-up period after a blanking period of 3 months. Patients underwent direct-current cardioversion if there was a sustained early recurring AF. Further antiarrhythmic drug treatment was prescribed according to the discretion of the treating physician.

### Genotyping

Genomic DNA was extracted from whole blood of AF patients using a DNA Extraction Kit (QIAamp DNA Blood Mini Kit), according to the manufacturer’s instructions. The tag-SNPs of *SCN10A* were selected using Han Chinese in Beijing (CHB) genotype data that was obtained from the International HapMap Project (http://hapmap.ncbi.nlm.nih.gov). To identify common tag-SNPs, the appropriate SNPs were entered into the Tagger programme and executed in the Haploview 4.2 programme (https://www.broadinstitute.org/haploview/downloads). Common variants were defined as those with a minor allele frequency (MAF) of more than 5% and set the threshold of 0.8 for the LD measure r^2^. In addition to rs6795970, 14 other tag-SNPs (i.e., rs6790627, rs4076737, rs12632942, rs7374804, rs7630989, rs62244070, rs6798015, rs7644332, rs4676596, rs10212338, rs11716467, rs11926158, rs9879472, and rs9827941) were selected for this study. These tag-SNPs were genotyped using iPLEX chemistry on a matrix-assisted laser desorption/ionization time-of-flight mass spectrometer (MALDI-TOF-MS, also known as a MassARRAY system; Sequenom, Inc.). The primers that were used for polymerase chain reaction (PCR) are shown in Supplementary Table S1. The specific genotyping protocol that was used in this study has been described in detail elsewhere^27^. All genotyping results were generated and verified by laboratory staff who were unaware of patient status.

### Statistical analysis

Continuous variables are expressed as the mean ± standard deviation (SD). Categorical variables are expressed as frequencies. Unpaired Student’s *t* test was used to compare continuous variables. Chi-square tests were used to compare categorical variables and performed to analyse the percentage of AF recurrence in each *SCN10A* genotype. Hardy-Weinberg equilibrium was also tested using Chi-square tests^28^. A p value < 0.05 was considered to indicate statistical significance. Three different genetic models (additive, dominant, and recessive) were performed to determine genotype-rhythm outcome associations. A multivariable logistic regression analysis was performed to evaluate the associations between *SCN10A* genotypes and AF recurrence, and the results were adjusted for clinical variables including sex, age (≥60 vs. <60 years old), AF subtype, history of AF (≥36 vs. <36 months), LAD (≥40 vs. <40 mm), LVEF, hypertension, coronary heart disease, rheumatic heart disease, cerebral embolism, diabetes, smoking, alcohol, linear ablations, CFAEs ablations, cardioversion during ablation and previous use of amiodarone, BB, CCB and ACEI/ARB. The cut-off value for LAD was set at 40 mm because the normal range of LAD was 19–40 mm. Because the mean duration of AF history was approximately 36 months, this length of time was set as the cut-off value for the history of AF. In addition, the cut-off value for age was set at 60 years old, as the mean age was approximately 60 years old. The LVEF was a continuous variable. There was no appropriate cut-off value for LVEF, as the vast majority of patients with LVEF were in the normal range (55–75%). Bonferroni correction was used to correct for multiple genetic tests of factors with a p value < 0.0033 (0.05/15), which was considered to indicate statistical significance. The test for the effect of rs6795970 on AF recurrence was hypothesis-driven, and the p value was not corrected for the number of comparisons. LD values (D’, r^2^ values) were estimated using the Haploview 4.2 programme^29^. Haplotype blocks were determined using the default method of the algorithm describe in Gabriel *et al*.^30^. Moreover, a haplotype analysis was performed using the HAPLO.STATS package^31^. A multivariable logistic regression analysis was also performed to evaluate the associations between each variant haplotype and AF recurrence. A stratified analysis was further performed by history of AF and AF subtype. In cases which genetics were missing, the missing data was handled by the method of “exclude cases analysis by analysis”. All tests were two-tailed, and all data were analysed using SPSS software version 19.0 (SPSS Inc., Chicago, IL, USA).

## Results

### Demographic and clinical characteristics

A total of 502 consecutive AF patients were enrolled in this study. AF recurrence was observed between 3 and 12 months after ablation in 24.5% (123/502) of the patients. All patients with AF recurrence were compared to those with no recurrence, as shown in Table 1. There were 331 paroxysmal and 171 non-paroxysmal AF patients, in which AF recurrence was observed between 3 and 12 months after ablation in 20.5% (68/331) and 32.2% (55/171) respectively. There were 287 males and the mean age of all patients was 60.1 years old. The mean duration for AF history was 35.9 months. A total of 273 patients presented with hypertension, and 30 patients presented with coronary heart disease. In all, 120 patients had previously received amiodarone, and 172 patients had previously received beta-receptor blocker drugs. In all, 297 patients underwent additional linear ablations and 107 patients underwent CFAEs ablations. A total of 95 patients underwent cardioversion during ablation. There were significant differences between the AF recurrence and no recurrence groups according to AF subtype (p = 0.004), history of AF (p < 0.001), LAD (p < 0.001), whether a CFAEs ablation was performed (p = 0.006), and whether cardioversion occurred during ablation (p = 0.004). However, there were no significant differences in other clinical factors among these two groups. Patients with AF recurrence compared with no recurrence in paroxysmal AF group and non-paroxysmal AF group was shown in Table 2 and Table 3. In both subgroups, there were significant differences between the AF recurrence and no recurrence groups according to history of AF and LAD.

A total of 33 (6.6%) complications were encountered in this study population. There were 12 complications related to vascular access (i.e., 6 femoral haematomas, 3 femoral arterio-venous fistulas and 3 femoral pseudo-aneurysms), and these were handled conservatively. Moreover, there were 3 pneumothorax and 4 cerebral infarctions, which were also handled conservatively and resulted in no sequelae. In addition, there were 8 pericardial effusions (all managed conservatively with no sequelae) and 6 pericardial tamponades (resolved using pericardiopuncture in 4 patients and surgical drainage in 2 patients). There were no other major complications.

### Association between *SCN10A* genotypes and AF recurrence

We selected 15 tag-SNPs in *SCN10A* according to the method described in Section 2. The relevant information related to these SNPs is presented in Supplementary Table S2. The genotyping rate ranged from 97.6% to 99.6%. There was no deviation from the Hardy-Weinberg equilibrium in any of these SNPs. Of these tag-SNPs, there was a significant difference between the AF recurrence and no recurrence groups in the allele distribution of rs6795970 (A vs. G, OR 0.41, 95%CI 0.26~0.65, p = 7.86 × 10^−5^). Additionally, there was a significant difference in the genotype distribution of rs6795970 between both groups (p = 1.42 × 10^−5^, Table 4). The rates of AF recurrence in patients with the GG, GA and AA genotypes were 30.5%, 11.9% and 20.0%, respectively. In a multivariate analysis of all AF patients (Table 4), rs6795970 genotypes were significantly associated with AF recurrence in both the additive model (OR 0.36, 95%CI 0.22~0.60, p = 7.04 × 10^−5^) and the dominant model (OR 0.29, 95%CI 0.16~0.50, p = 9.31 × 10^−6^) after adjustment for other clinical variables. The rs6795970 genotypes for AA vs. GA vs. GG (OR 0.36, 95%CI 0.22~0.60, p = 7.04 × 10^−5^), a history of AF ≥36 months (OR 3.57, 95%CI 2.26~5.63, p = 4.33 × 10^−8^) and LAD ≥40 mm (OR 1.85, 95%CI 1.08~3.19, p = 0.026) were independent predictors of AF recurrence (Table 5). However, there was no significant association between the other 14 tag-SNPs and AF recurrence in any of the three different genetic models that were evaluated (see Supplementary Table S3).

In the history of AF ≥36 months subgroup, after adjustment, there was a significant relationship between rs6795970 genotypes and AF recurrence in the additive model (OR 0.33, 95%CI 0.17~0.66, p = 0.002) and the dominant model (OR 0.30, 95%CI 0.14~0.63, p = 0.002). In addition, in the analysis of the history of AF <36 months subgroup, the rs6795970 genotypes were associated with AF recurrence in the additive model (OR 0.43, 95%CI 0.21~0.86, p = 0.018) and the dominant model (OR 0.32, 95%CI 0.15~0.69, p = 0.004) after adjustment. In the multivariate analysis of the paroxysmal and non-paroxysmal AF subgroups, the rs6795970 genotypes were also related to AF recurrence in two genetic models (additive and dominant) after adjustment (Table 4).

### Linkage disequilibrium (LD) and block analysis of 15 tag-SNPs in *SCN10A*

**Figure 1:**
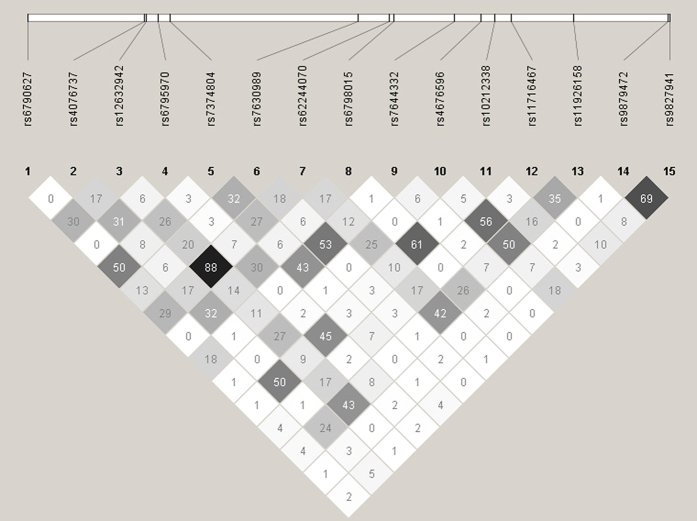
Graphical representation of the linkage disequilibrium (LD) structure in the *SCN10A* gene region. This figure shows a graph that represents measurements of LD values (r^2^) among all possible pairs of SNPs. LD values are expressed as differences in the shade of each colour. White represents a very low r^2^, and scarlet represents a very high r^2^. The numbers within the squares represent the r^2^ values (r^2^ × 100).

**Figure 2:**
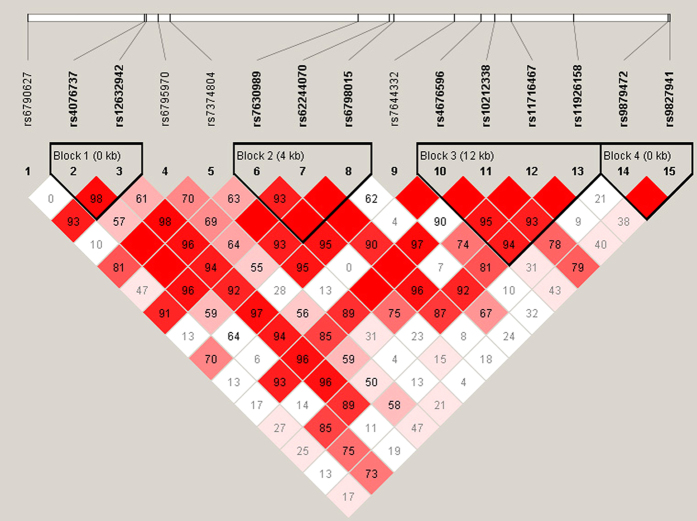
Graphical representation of the haplotype block structure in the *SCN10A* gene region. This figure shows a graph that represents measurements of LD values (D’) among all possible pairs of SNPs. LD values are indicated as differences in the shade of each colour. White represents a very low D’, and red represents a very high D’. The numbers within the squares represent the D’ values (D’ × 100). The LD plot was generated using the Haploview programme and the genotype data that were obtained during this study. Haplotype blocks were determined using the default method described in the Gabriel et al. algorithm, and four blocks were identified in this study.

The calculations of the pairwise LD (r^2^) values for these tag-SNPs are shown in Fig. 1, and the plots of the pairwise LD (D’) values for the tag-SNPs and the LD structures for each gene are shown in Fig. 2. There was almost no strong LD between these SNPs, suggesting they may independently contribute to the association. Because all of these SNPs were located in the *SCN10A* gene region, we next performed a haplotype analysis. Haplotype blocks were plotted according to previously published criteria^30^, and four blocks were identified (Fig. 2). Block 1 was formed by rs4076737 and rs12632942, block 2 was formed by rs7630989, rs62244070 and rs6798015, block 3 was formed by rs4676596, rs10212338, rs11716467 and rs11926158, and block 4 was formed by rs9879472 and rs9827941. In the multivariate analysis, there was no significant association between the *SCN10A* haplotypes and AF recurrence (see Supplementary Table S4).

## Discussion

In the current study, we analysed the genotypes of 15 tag-SNPs in *SCN10A* in 502 AF patients who underwent catheter ablation. These patients were monitored for 12 months to detect AF recurrence. The results showed that a patient’s rs6795970 genotypes were associated with the outcome of AF recurrence after ablation, confirming our original hypothesis. It is worth noting that the association between the rs6795970 genotypes and AF recurrence was independent of other clinical factors. Moreover, a longer history of AF and left atrial enlargement both contributed to AF recurrence, consistent with the result of previous studies^6^,^32^. We also performed a stratified analysis by history of AF and AF subtype. The rs6795970 genotypes were independently associated with AF recurrence in these subgroups. In addition, we performed a LD and haplotype block analysis that identified four blocks in *SCN10A*. To the best of our knowledge, this study is the first to investigate the relationship between *SCN10A* polymorphisms and AF recurrence after ablation in a Chinese Han population. Our research findings demonstrate that genetic factors play an important role in the efficacy of AF ablation.

AF is the most commonly sustained arrhythmia and has been independently associated with an increased risk of stroke, heart failure and death^1^. The prevalence of AF increases dramatically with age^33^. A number of common genetic risk factors have been reported to associate with AF, including variants on chromosome 4q25 near *PITX2*, *KCNH2*, *ACE*, *SCN5A* and *SCN10A*^13^,^16^,^34^,^35^,^36^. *SCN10A* polymorphisms have been shown to be associated with AF^16^,^17^,^37^. Pfeufer *et al*.^17^ demonstrated that rs6800541 was significantly associated with AF and was in strong LD with rs6795970. Ritchie *et al*.^16^ showed that the G allele of rs6795970 was associated with a higher risk of AF. In addition, Jabbari *et al*.^37^ also reported that the G allele of rs6795970 increased the risk of AF. Our data demonstrate that the G allele of rs6795970 increases the AF recurrence rate after catheter ablation. The direction of association is the same as in the association with AF previously reported as in the association with AF recurrence after catheter ablation shown in this study. It is worth noting that the magnitude of the effect on AF recurrence after ablation was much greater than the magnitude of the reported effect on AF in Europeans. In future studies, we will further investigate the effect of rs6795970 polymorphisms on AF and AF recurrence after ablation in a second population to confirm our finding.

It has also been previously shown that some genetic factors predict the risk of AF recurrence after catheter ablation^18^,^19^,^20^,^21^,^22^,^23^. Recently, Husser *et al*.^18^ showed that noncoding variants on chromosome 4q25, including rs2200733 and rs10033464, influence the risk of AF recurrence after ablation. Shoemaker *et al*.^19^ reported that rs2200733 was significantly associated with AF recurrence after ablation. Wutzler *et al*.^21^ found that rs751141 in cytoplasmic epoxide hydrolase 2 (*EPHX2)* was associated with a significantly higher risk of AF recurrence after ablation. Hu *et al*.^20^ showed that haeme oxygenase-1 (*HO-1)* gene promoter polymorphisms were associated with AF recurrence after ablation. Ueberham *et al*.^22^ demonstrated that *ACE* DD polymorphisms and left atrial enlargement were independent predictors of AF recurrence after ablation. Wu *et al*.^23^ reported that rs4845625 in *IL6R* contributed to AF recurrence after ablation in a Chinese Han population. Finally, our results revealed that there is an association between rs6795970 and AF recurrence after ablation in a Chinese Han population.

We describe a previously unreported association between *SCN10A* polymorphisms and treatment response after catheter ablation in AF patients. However, the precise mechanism underlying this relationship remains to be determined. Autonomic innervations of the heart include both the extrinsic and intrinsic cardiac autonomic nervous system (ECANS and ICANS, repectively). The ICANS forms a complex neural network that consists of ganglionated plexi (GP), which are concentrated within epicardial fat pads^38^,^39^. It was previously demonstrated that GP play an essential role in the initiation and maintenance of AF^40^,^41^,^42^. Hence, GP ablation could be used as a supplement for conventional PV isolation in patients undergoing AF ablation, as has been reported in some clinical trials^43^,^44^. *SCN10A* encodes Na_v_1.8, an alpha subunit of Na_v_. Na_v_1.8, a TTX-resistant sodium channel, is highly expressed in the small-diameter sensory neurons of dorsal root ganglia. Na_v_1.8 is known to play a critical role in generating and maintaining action potentials in nociceptive nerve fibres^11^,^45^. The human *SCN10A* gene is located on chromosome 3p22.2 and includes 27 exons. Recent studies have suggested that the impact of Na_v_1.8 on cardiac electrophysiological properties is mediated by its effects on intrinsic cardiac ganglia neurons. Facer *et al*.^46^ showed that Na_v_1.8-immunoreactive sensory nerve fibres are present in human atrial myocardium. Moreover, Verkerk *et al*.^8^ also demonstrated that *SCN10A*/Na_v_1.8 exist in intrinsic cardiac neurons. Immunocytochemical studies have further revealed that a substantial amount of Na_v_1.8 is expressed in isolated intrinsic cardiac ganglia neurons. However, no Na_v_1.8 is expressed in isolated ventricular myocytes. In a recent study, Qi *et al*.^47^ demonstrated that blocking Na_v_1.8 channels inhibited the effects of vagus nerve stimulation on cardiac conduction and AF inducibility, likely by suppressing the neural activity of the cardiac GP. It has been suggested that *SCN10A*/ Na_v_1.8 play a functional role in modulating AF inducibility. It is likely that Na_v_1.8 channels accommodate the release of neurotransmitters and/or neuropeptides in GP and that acetylcholine and NO may be the factors that mediate these effects. Blasius *et al*.^48^ reported that mice carrying the *SCN10A* Possum mutation, which strengthens Na_v_1.8 sodium currents and neuronal excitability, respond to ‘scruffing’ with distinct sinus bradycardia and R-R variability and that these effects could be blocked by an infusion of atropine. Herring *et al*.^49^ demonstrated that NO increased the induced release of acetylcholine and the vagus nerve-mediated heart rate response by affecting a pre-synaptic pathway that involves phosphodiesterase 3 and protein kinase A.

The *SCN5A* gene is a major cardiac sodium channel that has mutations known to lead to long-QT syndrome, Brugada syndrome, cardiac conduction disease and AF^50^. The rs6795970 is located 75.5 kb away from *SCN5A*. Data from HapMap revealed that rs6795970 is not associated with any common sequence variant of *SCN5A* at r^2^ > 0.01 in a Han Chinese Beijing populations. Moreover, several studies have demonstrated that rs6795970 is not in LD with common *SCN5A* polymorphisms (i.e., rs12053903 and rs7638909) that have been associated with cardiac conduction^51^,^52^,^53^. These data showed that the association between rs6795970 and AF recurrence is not mediated through *SCN5A*. The rs6795970 (G > A) is a missense mutation that causes an A1073V amino acid change within the IDII/III intracellular loop of Na_v_1.8^13^. The cytoplasmic domains of sodium channels regulate channel function via cAMP-dependent phosphorylation and interactions with accessory proteins including PDZD2^54^,^55^. Recently, it was shown that single amino acid changes within the IDII/III loop of other sodium channels are implicated in abnormalities in cardiac conduction and altered electrophysiological properties^55^. Therefore, the fact that rs6795970 (G > A) results in an amino acid change suggests that it could influence the outcome of ablations in AF patients. However, the mechanisms underlying its effects will require more in-depth investigations.

In this study, we provide new insights that may lead a novel approach to ameliorating the therapeutic effects of AF ablations, especially in patients with specific *SCN10A* genotypes, such as the rs6795970 GG genotype. *SCN10A* genotypes should be considered along with other previously reported efficacy predictors when evaluating potential outcomes in AF patients who undergo catheter ablations.

There are several limitations to this study. First, this was a retrospective study that included a relatively limited sample size. To confirm these findings, we will include a large cohort of patients in future studies. Second, although a 24-hour Holter monitor is an effective way to recognize frequent asymptomatic recurrences, a few asymptomatic paroxysmal arrhythmia recurrences may have been missed. Therefore, we may have underestimated the true rate of AF recurrence. If 7-day Holter monitor or event recorders were used during the follow-up period, we may have achieved more accurate results. Third, the follow-up period was limited to 12 months. Hence, it is possible that very late AF recurrences were not included in the analysis. Moreover, our selection criteria limited the study to AF patients in a Chinese Han population. Therefore, it remains unknown whether our research findings can be extended to other populations.

In conclusion, our data suggest that the rs6795970 genotypes in *SCN10A* are independently associated with AF recurrence in Chinese Han patients who undergo a catheter ablation. This finding suggests a potential role for stratifying the ablation procedure or post-ablation management according to genotype.

## Additional Information

**How to cite this article:** Wu, H. *et al*. Association of *SCN10A* Polymorphisms with the Recurrence of Atrial Fibrillation after Catheter Ablation in a Chinese Han Population. *Sci. Rep.* **7**, 44003; doi: 10.1038/srep44003 (2017).

**Publisher's note:** Springer Nature remains neutral with regard to jurisdictional claims in published maps and institutional affiliations.

## Supplementary Material

Supplementary Information

## Figures and Tables

**Table 1:** Comparison of patient characteristics with and without AF recurrence in all AF patients.

	No recurrence n = 379	AF recurrence n = 123	P
Male, n (%)	219 (57.8)	69 (56.1)	0.742
Age, years old	59.9 ± 10.0	60.7 ± 9.9	0.434
Paroxysmal AF, n (%)	263 (69.4)	68 (55.3)	0.004
Non-paroxysmal AF, n (%)	116 (30.6)	55 (44.7)	0.004
History of AF, months	33.2 ± 25.6	44.0 ± 22.8	<0.001
LAD, mm	39.8 ± 5.4	42.5 ± 5.7	<0.001
LVEF, %	63.8 ± 5.5	62.5 ± 7.1	0.063
Hypertension, n (%)	210 (55.4)	63 (51.2)	0.418
Coronary heart disease, n (%)	23 (6.1)	7 (5.7)	0.878
Rheumatic heart disease, n (%)	5 (1.3)	4 (3.3)	0.311
Cerebral embolism, n (%)	20 (5.3)	6 (4.9)	0.862
Diabetes, n (%)	48 (12.7)	24 (19.5)	0.060
Smoking, n (%)	61 (16.1)	17 (13.8)	0.545
Alcohol, n (%)	30 (7.9)	11 (8.9)	0.718
Amiodarone use, n (%)	89 (23.5)	31 (25.2)	0.697
BB use, n (%)	128 (33.8)	44 (35.8)	0.685
CCB use, n (%)	47 (12.4)	14 (11.4)	0.764
ACEI/ARB use, n (%)	136 (35.9)	40 (32.5)	0.497
Linear ablations, n (%)	219 (57.8)	78 (63.4)	0.270
CFAEs ablations, n (%)	70 (18.5)	37 (30.1)	0.006
Cardioversion during ablation, n (%)	61 (16.1)	34 (27.6)	0.004

Note: AF: atrial fibrillation; LAD: left atrial diameter; LVEF: left ventricular ejection fraction; BB: beta-receptor blocker; CCB: calcium channel blocker; ACEI: angiotensin-converting enzyme inhibitor; ARB: angiotensin II receptor blocker. CFAEs: complex fractionated atrial electrograms.

**Table 2:** Comparison of patient characteristics with and without AF recurrence in paroxysmal AF group.

	No recurrence n = 263	AF recurrence n = 68	P
Male, n (%)	137 (52.1)	33 (48.5)	0.600
Age, years old	60.6 ± 10.3	61.8 ± 9.4	0.399
History of AF, months	32.5 ± 24.8	42.0 ± 18.0	0.001
LAD, mm	38.3 ± 5.0	40.4 ± 4.9	0.003
LVEF, %	64.8 ± 4.5	63.3 ± 7.2	0.104
Hypertension, n (%)	149 (56.7)	38 (55.9)	0.909
Coronary heart disease, n (%)	21 (8.0)	5 (7.4)	0.863
Rheumatic heart disease, n (%)	3 (1.1)	2 (2.9)	0.598
Cerebral embolism, n (%)	17 (6.5)	4 (5.9)	1.000
Diabetes, n (%)	33 (12.5)	14 (20.6)	0.090
Smoking, n (%)	36 (13.7)	10 (14.7)	0.829
Alcohol, n (%)	14 (5.3)	5 (7.4)	0.727
Amiodarone use, n (%)	62 (23.6)	17 (25.0)	0.806
BB use, n (%)	90 (34.2)	25 (36.8)	0.695
CCB use, n (%)	33 (12.5)	8 (11.8)	0.861
ACEI/ARB use, n (%)	95 (36.1)	22 (32.4)	0.562
Linear ablations, n (%)	116 (44.1)	27 (39.7)	0.514
CFAEs ablations, n (%)	5 (1.9)	4 (5.9)	0.167
Cardioversion during ablation, n (%)	3 (1.1)	1 (1.5)	1.000

Note: AF: atrial fibrillation; LAD: left atrial diameter; LVEF: left ventricular ejection fraction; BB: beta-receptor blocker; CCB: calcium channel blocker; ACEI: angiotensin-converting enzyme inhibitor; ARB: angiotensin II receptor blocker. CFAEs: complex fractionated atrial electrograms.

**Table 3:** Comparison of patient characteristics with and without AF recurrence in non-paroxysmal AF group.

	No recurrence n = 116	AF recurrence n = 55	P
Male, n (%)	82 (70.7)	36 (65.5)	0.489
Age, years old	58.1 ± 9.1	59.3 ± 10.2	0.460
History of AF, months	34.7 ± 27.5	46.4 ± 27.6	0.010
LAD, mm	43.1 ± 4.9	45.1 ± 5.5	0.015
LVEF, %	61.3 ± 6.7	61.4 ± 6.9	0.962
Hypertension, n (%)	61 (52.6)	25 (45.5)	0.384
Coronary heart disease, n (%)	2 (1.7)	2 (3.6)	0.817
Rheumatic heart disease, n (%)	2 (1.7)	2 (3.6)	0.817
Cerebral embolism, n (%)	3 (2.6)	2 (3.6)	1.000
Diabetes, n (%)	15 (12.9)	10 (18.2)	0.364
Smoking, n (%)	25 (21.6)	7 (12.7)	0.167
Alcohol, n (%)	16 (13.8)	6 (10.9)	0.599
Amiodarone use, n (%)	27 (23.3)	14 (25.5)	0.755
BB use, n (%)	38 (32.8)	19 (34.5)	0.817
CCB use, n (%)	14 (12.1)	6 (10.9)	0.826
ACEI/ARB use, n (%)	41 (35.3)	18 (32.7)	0.737
Linear ablations, n (%)	103 (88.8)	51 (92.7)	0.422
CFAEs ablations, n (%)	65 (56.0)	33 (60.0)	0.624
Cardioversion during ablation, n (%)	58 (50.0)	33 (60.0)	0.221

Note: AF: atrial fibrillation; LAD: left atrial diameter; LVEF: left ventricular ejection fraction; BB: beta-receptor blocker; CCB: calcium channel blocker; ACEI: angiotensin-converting enzyme inhibitor; ARB: angiotensin II receptor blocker. CFAEs: complex fractionated atrial electrograms.

**Table 4:** Association between rs6795970 genotypes and AF recurrence in three different genetic models.

Group	Genotype	No recurrence, n%	AF recurrence, n%	Recurrence rate (%)	P^a^	Additive model (adjusted) (AA vs.GA vs. GG)		Dominant model (adjusted) (AA + GA vs. GG)		Recessive model (adjusted) (AA vs. GG + GA)	
						OR (95%CI)	P^b^	OR(95%CI)	P^b^	OR (95%CI)	P^b^
All AF patients	GG	226 (59.6)	99 (81.8)	30.5	1.42 × 10^-5^	0.36 (0.22~0.60)	7.04 × 10^-5^	0.29 (0.16~0.50)	9.31 × 10^−6^	0.82 (0.21~3.24)	0.780
	GA	141 (37.2)	19 (15.7)	11.9							
	AA	12 (3.2)	3 (2.5)	20.0							
History of AF ≥36 months group	GG	62 (58.5)	58 (82.9)	48.3	0.002	0.33 (0.17~0.66)	0.002	0.30 (0.14~0.63)	0.002	0.29 (0.03~2.68)	0.274
	GA	38 (35.8)	11 (15.7)	22.4							
	AA	6 (5.7)	1 (1.4)	14.3							
History of AF <36 months group	GG	164 (60.1)	41 (80.4)	20.0	0.004	0.43 (0.21~0.86)	0.018	0.32 (0.15~0.69)	0.004	2.02 (0.37~11.07)	0.419
	GA	103 (37.7)	8 (15.7)	7.2							
	AA	6 (2.2)	2 (3.9)	25.0							
Paroxysmal AF group	GG	154 (58.6)	53 (79.1)	25.6	0.005	0.41 (0.21~0.78)	0.007	0.32 (0.16~0.66)	0.002	0.97 (0.18~5.32)	0.974
	GA	100 (38.0)	12 (17.9)	10.7							
	AA	9 (3.4)	2 (3.0)	18.2							
Non-paroxysmal AF group	GG	72 (62.1)	46 (85.2)	39.0	0.004	0.34 (0.16~0.75)	0.007	0.28 (0.12~0.66)	0.004	0.80 (0.07~9.62)	0.860
	GA	41 (35.3)	7 (13.0)	14.6							
	AA	3 (2.6)	1 (1.9)	25.0							

Note: AF: atrial fibrillation. a: P values were calculated from a case-control analysis using the Chi-square test. b: P values were calculated by a multivariable logistic regression analysis after adjustment for clinical variables including sex, age (≥60 vs. <60 years old), AF subtype, history of AF (≥36 vs. <36 months), LAD (≥40 vs. <40 mm), LVEF, hypertension, coronary heart disease, rheumatic heart disease, cerebral embolism, diabetes, smoking, alcohol, linear ablations, CFAEs ablations, cardioversion during ablation and previous use of amiodarone, BB, CCB and ACEI/ARB.

**Table 5:** Multivariate analysis of predictors for AF recurrence in rs6795970 genotypes in an additive model.

	Multivariate analysis	
	OR (95% CI)	P
rs6795970 genotype (AA vs. GA vs. GG)	0.36 (0.22~0.60)	7.04 × 10^−5^
Sex (female vs. male)	1.07 (0.64~1.78)	0.798
Age (≥60 vs. <60 years old)	1.05 (0.65~1.70)	0.829
AF subtype (paroxysmal vs. non-paroxysmal)	0.79 (0.38~1.67)	0.543
History of AF (≥36 vs. <36 months)	3.57 (2.26~5.63)	4.33 × 10^−8^
LAD (≥40 vs. <40 mm)	1.85 (1.08~3.19)	0.026
LVEF	0.98 (0.94~1.01)	0.191
Hypertension (with vs. without)	0.75 (0.47~1.21)	0.244
Coronary heart disease (with vs. without)	0.83 (0.31~2.21)	0.703
Rheumatic heart disease (with vs. without)	2.03 (0.46~8.94)	0.348
Cerebral embolism (with vs. without)	0.57 (0.19~1.74)	0.323
Diabetes (with vs. without)	1.85 (1.00~3.42)	0.051
Smoking (with vs. without)	0.57 (0.19~1.74)	0.171
Alcohol (with vs. without)	1.69 (0.63~4.53)	0.296
Amiodarone use (with vs. without)	1.00 (0.59~1.70)	0.997
BB use (with vs. without)	1.01 (0.63~1.64)	0.956
CCB use (with vs. without)	0.91 (0.44~1.88)	0.800
ACEI/ARB use (with vs. without)	0.93 (0.57~1.50)	0.764
Linear ablations (with vs. without)	0.78 (0.46~1.33)	0.361
CFAEs ablations (with vs. without)	1.38 (0.69~2.78)	0.365
Cardioversion during ablation (with vs. without)	1.88 (0.89~3.95)	0.097

Note: AF: atrial fibrillation; LAD: left atrial diameter; LVEF: left ventricular ejection fraction; BB: beta-receptor blocker; CCB: calcium channel blocker; ACEI: angiotensin-converting enzyme inhibitor; ARB: angiotensin II receptor blocker. CFAEs: complex fractionated atrial electrograms.
