# Supplementary material for: Association of SCN10A Polymorphisms with the Recurrence of Atrial Fibrillation after Catheter Ablation in a Chinese Han Population
Source: Sci Rep. 2017 Mar 10;7:44003. doi: 10.1038/srep44003 (PMC5345091; doi:10.1038/srep44003)
Supplement: Supplementary Information [file srep44003-s1.pdf]

Supplementary Tables

Association of *SCN10A* Polymorphisms with the Recurrence of Atrial Fibrillation after Catheter Ablation in a Chinese Han Population

Haiqing Wu[1+], Juan Xu[1+], Songwen Chen[1+], Liqun Zhao[1+], Genqing Zhou[1], Baozhen Qi[2], Yong Wei[3], En Hu[1], Dongdong Tang[1], Gang Chen[1], Hongli Li[1], Yongyong Shi[4*], Shaowen Liu[1*]

[1]Department of Cardiology, Shanghai First People's Hospital, Shanghai Jiao Tong University School of Medicine, Shanghai, 200080, China

[2]Department of Cardiology, Zhongshan Hospital, Fudan University, Shanghai, 200032, China.

[3]Department of Cardiology, Shanghai Songjiang Center Hospital, Shanghai, 201600, China

[4]Bio-X Institutes, Key Laboratory for the Genetics of Developmental and Neuropsychiatric Disorders (Ministry of Education), Shanghai Jiao Tong University, Shanghai, 200030, China

[+]The authors contribute equally to this work

[*]Corresponding author e-mails:

Professor Shaowen Liu, shaowen.liu@hotmail.com.

Professor Yongyong Shi, shiyongyong@gmail.com.

Supplementary Table S1. The primers used for polymerase chain reaction

SNP ID	Forward primer sequence	Reverse primer sequence	Extended primer sequence
rs6790627	ACGTTGGATGTCTTGGAG CCCAACTTCTTC	ACGTTGGATGTTAGTAAC AGTGTGGCCCTC	GACAGAGGAGCAGAA GAAA
rs4076737	ACGTTGGATGAACAAGCT GTGCCCACTTGC	ACGTTGGATGATGAGATG CAGATACCACGG	GCATGTGTGTCTGATG CG
rs12632942	ACGTTGGATGTCTGGTTC TTCCAGGTCATC	ACGTTGGATGTGGACGAC ACAAGCTCCTCT	cgaAGCACGGTGGACT GCCC
rs6795970	ACGTTGGATGGCACTGGA ACATCTTCTGAG	ACGTTGGATGAGGAAGCT GACATACCTACC	CCTACCTCAGCAGGG A
rs7374804	ACGTTGGATGTGGTTCTC AGCCTTGGAGC	ACGTTGGATGTGCAGCTT CTTCAGCAGGTC	gtTGAGCTGGTGGTGA AA
rs7630989	ACGTTGGATGCTAGTCAT GGCAGTGTGTTC	ACGTTGGATGCCAGGAAA GACTCCATCATC	TATCTCGGCCAGGGG A
rs62244070	ACGTTGGATGCTTCACAA AGCTTCCACTCC	ACGTTGGATGAGGAGAAG AAGTTCCAGGAG	gggagCGAGATGCTCCG GAAGGAA
rs6798015	ACGTTGGATGGATCTTGG ACACTTGGTCTG	ACGTTGGATGCTCCCCTG TGTATGAAATTC	gaggCTGACGTGAAAG TATTAGCA
rs7644332	ACGTTGGATGATCTGGAG GCATCACCTTAG	ACGTTGGATGCCATTGGT CTATGTGCTTAT	aagtcTCATGCTGTTTTG GTACAA
rs4676596	ACGTTGGATGCAGAAGGC TGATTATTAAGTG	ACGTTGGATGTCTCTGGA GAATTTTCCATC	tgtTTTTTATCTTTCTCT GGTGTCTC

rs10212338	ACGTTGGATGCTGGTATATTCCTGCAGTGG	ACGTTGGATGTAACATGCAGGTCCCACTTG	tGAGACTCACACCATGAAC
rs11716467	ACGTTGGATGTTTGCCTCTTCCCTGGATCG	ACGTTGGATGGGCAGCATAGAGAGACTTTG	TGCTCCATTCAAACAAGA
rs11926158	ACGTTGGATGTGGTCTGAAACCCCAATTAC	ACGTTGGATGACTGGAAGAATTGTACATC	ATCAAAAAATAAAAAGCAAATAAGAC
rs9879472	ACGTTGGATGATGGTATCCCTAGTCAGCAG	ACGTTGGATGTCATTGGCTTACTGCAAGGG	GACAGGGAATAGTGAGAAAAC
rs9827941	ACGTTGGATGGGGAAAGGAATGGTCCTTAG	ACGTTGGATGTCAAACCTCCAACTCACCTG	CTTACAACATGAGAGGTATCA

Supplementary Table S2. Characteristics of 15 tag-SNPs in *SCN10A*

No	SNP ID	Chromosome position[a]	Genic location	Base change	P[b]	OR (95%CI)	P[c]	Genotyping rate (%)
1	rs6790627	38707342	exon	C/T	0.560	1.10 (0.80~1.50)	0.474	98.2
2	rs4076737	38723291	intron	C/A	0.893	0.97 (0.67~1.41)	0.765	98.0
3	rs12632942	38723507	exon	G/A	0.989	1.00 (0.75~1.34)	0.560	98.2
4	rs6795970	38725184	exon	A/G	7.86×10^{-5}	0.41 (0.26~0.65)	0.072	99.6
5	rs7374804	38726843	exon	C/T	0.623	1.09 (0.78~1.51)	0.863	97.8
6	rs7630989	38752449	exon	G/A	0.439	0.87 (0.61~1.24)	0.105	98.6
7	rs62244070	38756680	exon	T/C	0.824	1.03 (0.77~1.38)	0.382	98.0
8	rs6798015	38757345	intron	C/T	0.706	1.07 (0.74~1.56)	0.069	98.0
9	rs7644332	38765609	intron	A/G	0.333	1.22 (0.81~1.83)	0.786	97.8
10	rs4676596	38769206	intron	A/G	0.616	0.92 (0.66~1.27)	0.285	98.0
11	rs10212338	38771159	intron	A/G	0.662	1.10 (0.71~1.71)	0.345	97.8
12	rs11716467	38773345	intron	G/A	0.876	1.03 (0.72~1.48)	0.071	98.0
13	rs11926158	38781824	intron	G/C	0.548	0.91 (0.68~1.23)	0.791	97.8
14	rs9879472	38794803	5'UTR	G/C	0.599	0.91 (0.63~1.30)	0.773	97.6
15	rs9827941	38794968	5'UTR	A/T	0.588	0.91 (0.66~1.27)	0.814	98.0

Note: a. chromosome position in the NCBI dbSNPs database (http://www.ncbi.nlm.nih.gov/snp); b. p value for difference in allele distributions between the AF recurrence group and no recurrence group using the Chi-square test; c. p value for the Hardy-Weinberg equilibrium test in the control group.

Supplementary Table S3. Association between 15 tag-SNPs of *SCN10A* genotypes and AF recurrence

No	SNP ID	Genotype	No recurrence, n%	AF recurrence, n%	P[a]	Additive model (adjusted)		Dominant model (adjusted)		Recessive model (adjusted)	
						OR (95%CI)	P[b]	OR(95%CI)	P[b]	OR (95%CI)	P[b]
1	rs6790627	TT	176 (47.3)	57 (47.1)	0.438	1.02 (0.73~1.44)	0.897	0.87 (0.55~1.35)	0.528	1.65 (0.80~3.39)	0.174
		TC	164 (44.1)	49 (40.5)							
		CC	32 (8.6)	15 (12.4)							
2	rs4076737	AA	242 (64.9)	77 (64.7)	0.919	1.03 (0.68~1.55)	0.888	1.11 (0.69~1.78)	0.666	0.60 (0.15~2.42)	0.475
		AC	118 (31.6)	39 (32.8)							
		CC	13 (3.5)	3 (2.5)							
3	rs12632942	AA	121 (32.4)	37 (30.8)	0.835	1.03 (0.75~1.42)	0.832	1.11 (0.69~1.78)	0.679	0.97 (0.54~1.72)	0.905
		AG	178 (47.7)	61 (50.8)							
		GG	74 (19.8)	22 (18.3)							

4	rs6795970	GG	226 (59.6)	99 (81.8)	$1.42×10^{-5}$	0.36 (0.22~0.60)	$7.04×10^{-5}$	0.29 (0.16~0.50)	$9.31×10^{-6}$	0.82 (0.21~3.24)	0.780
		GA	141 (37.2)	19 (15.7)							
		AA	12 (3.2)	3 (2.5)							
5	rs7374804	TT	206 (55.7)	65 (53.7)	0.867	1.05 (0.73~1.51)	0.783	1.00 (0.63~1.56)	0.988	1.37 (0.58~3.24)	0.474
		TC	141 (38.1)	47 (38.8)							
		CC	23 (6.2)	9 (7.4)							
6	rs7630989	AA	221 (59.1)	76 (62.8)	0.799	0.80 (0.53~1.20)	0.284	0.77 (0.49~1.22)	0.263	0.84 (0.22~3.16)	0.792
		AG	140 (37.4)	42 (34.7)							
		GG	13 (3.5)	3 (2.5)							
7	rs62244070	CC	123 (33.2)	37 (30.6)	0.794	1.05 (0.77~1.44)	0.756	1.16 (0.72~1.87)	0.553	0.96 (0.54~1.70)	0.878
		CT	174 (46.9)	61 (50.4)							
		TT	74 (19.9)	23 (19.0)							
8	rs6798015	TT	257 (68.9)	76 (63.9)	0.131	1.04 (0.70~1.55)	0.857	1.24 (0.78~1.98)	0.368	0.28 (0.06~1.34)	0.112

		TC	99 (26.5)	41 (34.5)								
		CC	17 (4.6)	2 (1.7)								
9	rs7644332	GG	278 (74.9)	84 (70.0)	0.495	1.15 (0.74~1.80)	0.537	1.24 (0.75~2.04)	0.397	0.66 (0.12~3.58)	0.627
		GA	87 (23.5)	34 (28.3)							
		AA	6 (1.6)	2 (1.7)							
10	rs4676596	GG	193 (51.9)	63 (52.5)	0.640	0.97 (0.68~1.38)	0.880	1.05 (0.67~1.65)	0.830	0.73 (0.31~1.71)	0.472
		GA	144 (38.7)	49 (40.8)							
		AA	35 (9.4)	8 (6.7)							
11	rs10212338	GG	290 (78.4)	91 (75.2)	0.492	1.10 (0.68~1.78)	0.689	1.20 (0.71~2.03)	0.506	0.42 (0.04~4.08)	0.451
		GA	73 (19.7)	29 (24.0)							
		AA	7 (1.9)	1 (0.8)							
12	rs11716467	AA	246 (66.0)	76 (63.9)	0.733	1.11 (0.75~1.63)	0.608	1.20 (0.74~1.94)	0.451	0.86 (0.30~2.52)	0.788
		AG	107 (28.7)	38 (31.9)							

		GG	20 (5.4)	5 (4.2)							
13	rs11926158	CC	127 (34.2)	40 (33.3)	0.323	0.92 (0.66~1.27)	0.594	1.08 (0.67~1.74)	0.742	0.63 (0.33~1.20)	0.160
		CG	178 (48.0)	65 (54.2)							
		GG	66 (17.8)	15 (12.5)							
14	rs9879472	CC	224 (60.5)	77 (64.2)	0.719	0.97 (0.66~1.43)	0.884	0.91 (0.57~1.44)	0.683	1.33 (0.48~3.69)	0.585
		CG	129 (34.9)	37 (30.8)							
		GG	17 (4.6)	6 (5.0)							
15	rs9827941	TT	189 (50.8)	62 (51.7)	0.615	0.97 (0.68~1.39)	0.861	1.03 (0.66~1.62)	0.893	0.73 (0.30~1.82)	0.502
		TA	151 (40.6)	51 (42.5)							
		AA	32 (8.6)	7 (5.8)							

Note: AF: atrial fibrillation. a. P values were calculated from a case-control analysis using the Chi-square test. b. P values were calculated by a multivariable logistic regression analysis after adjustment for clinical variables including sex, age (≥60 vs. <60 years old), AF subtype, history of AF (≥36 vs. <36 months), LAD (≥40 vs. <40 mm), LVEF, hypertension, coronary heart disease, rheumatic heart disease, cerebral embolism,

diabetes, smoking, alcohol, linear ablations, CFAEs ablations, cardioversion during ablation and previous use of amiodarone, BB, CCB and ACEI/ARB.

Supplementary Table S4. Associations between SNPs of *SCN10A* haplotypes and AF recurrence

Block	Haplotype	No recurrence n (%)	AF recurrence n (%)	P[a]	P[b]	OR (95% CI)
Block 1	rs4076737-rs12632942					
	AG	331 (43.7)	108 (43.9)	0.968		1.00 (referent)
	AA	283 (37.3)	92 (37.4)	0.974	0.850	0. 97 (0.69-1.35)
	CA	143 (18.9)	46 (18.7)	0.962	0.995	1.00 (0.66-1.51)
	CG	1 (0.1)	0 (0.0)	N/A[c]	N/A[c]	N/A[c]
Block 2	rs7630989-rs62244070-rs6798015					
	ATT	326 (43.0)	108 (43.9)	0.790		1.00 (referent)
	GCT	163 (21.5)	47 (19.1)	0.435	0.390	0.84 (0.56-1.26)
	ACC	134 (17.7)	46 (18.7)	0.699	0.983	1.00 (0.66-1.53)
	ACT	132 (17.4)	44 (17.9)	0.920	0.931	1.02 (0.67-1.56)
	GTT	3 (0.4)	1 (0.4)	N/A[c]	N/A[c]	N/A[c]
Block 3	rs4676596-rs10212338-rs11716467-rs11926158					
	GGAC	433 (57.1)	142 (57.7)	0.871		1.00 (referent)
	AGGG	144 (19.0)	46 (18.7)	0.899	0.940	0.99 (0.66-1.47)
	GAAG	89 (11.7)	28 (11.4)	0.908	0.804	0.94 (0.58-1.53)
	AGAG	66 (8.7)	15 (6.1)	0.198	0.231	0.69 (0.37-1.27)
	Others	26 (3.4)	15 (6.1)	N/A[c]	N/A[c]	N/A[c]

Block 4	rs9879472-rs9827941					
	CT	543 (71.6)	181 (73.6)	0.556		1.00 (referent)
	GA	165 (21.8)	49 (19.9)	0.541	0.618	0.91 (0.62-1.32)
	CA	50 (6.6)	16 (6.5)	0.956	0.978	1.01 (0.54-1.87)

Note: a. P values for difference in haplotype frequency between AF recurrence and no recurrence group were calculated from a case-control analysis using the Chi-square test. b. P values were calculated by a multivariable logistic regression analysis after adjustment. c. N/A, not available because of the rarity (<3%) of haplotype.
